# Photon-pair generation in a lossy waveguide: Propagation loss effects on photon-pair generation in a lossy waveguide

**DOI:** 10.1515/nanoph-2022-0582

**Published:** 2023-01-10

**Authors:** Woncheol Shin, Kyungdeuk Park, Hyeongpin Kim, Dongjin Lee, Kiwon Kwon, Heedeuk Shin

**Affiliations:** Department of Physics, Pohang University of Science and Technology (POSTECH), Pohang, 37673, Korea

**Keywords:** coincidence-to-accidental ratio, photon-pair generation, propagation loss, spontaneous four-wave mixing

## Abstract

An on-chip quantum light source based on spontaneous four-wave mixing is an essential element for developing quantum photonic integrated circuit technology, which has the advantage of no connection loss owing to the integration of the source into photonic circuits. The waveguide-based quantum light source inevitably causes propagation loss owing to imperfections in the fabrication process, but the propagation loss effects on photon-pair generation have not been extensively studied. In this study, propagation loss effects were examined using theoretical and experimental methods. In theory, the performance of quantum light sources, such as brightness, heralding efficiency, and coincidence-to-accidental ratio, strongly depend on propagation loss. We fabricate several waveguides with a moderate propagation loss of 2.2 dB/cm to investigate the loss dependence and ascertain that the brightness, heralding efficiency, and coincidence-to-accident ratio strongly correlate with the length of the optical waveguide. The maximum coincidence-count brightness occurred at an optimization length of 1/*α*, where *α* is the absorption coefficient. In contrast, the single-count brightness shows slightly different waveguide length dependence owing to loss-induced one-photon states. We expect that the results obtained in this study will greatly assist in determining the proper waveguide length for photon-pair generation according to the source’s application fields. The results will be helpful in the development of a quantum light source suitable for practical and quantum optical integrated circuits and will lead to the development of high-fidelity quantum technologies.

## 1 Introduction

Quantum photonic integrated circuits (QPICs) have been extensively investigated, paving the way for innovative quantum technology applications [1]. With well-developed complementary metal-oxide semiconductor manufacturing technologies, QPICs have significant advantages over free-space and fiber-based quantum optical systems, including their size, stability, speed, and scalability [2, 3].

The QPIC system losses induced by imperfect detector efficiency, propagation loss, photonic device loss, and connection loss are significant obstacles to reducing quantum bit errors and increasing the information processing speed. The connection loss is significant when an external quantum light source is used [4–7]. Spontaneous four-wave mixing (SFWM) allows photon pair generation within the same materials as QPIC systems, yielding seamless integration between quantum light sources and systems [8, 9]. The reported waveguide structures for SFWM are designed to achieve the desired dispersion properties of phase-matching conditions [10–12]. The typical structure of an SFWM silicon waveguide for photon-pair generation near 1550 nm is a ridge-type waveguide with a narrow waveguide width that yields a zero-group-velocity dispersion (ZGVD) wavelength near the telecom range [13, 14]. However, the large scattering loss caused by sidewall roughness is significant in such a narrow waveguide structure [15, 16].

This propagation loss effect on photon pair generation has not been intensively investigated, as the propagation loss of a quantum light source can be simplified as a lump-sum loss. However, as propagation loss occurs throughout a waveguide, each section of the silicon waveguide can generate and lose photons. The propagation loss directly or indirectly affects the performance of quantum light sources, including pair-generation brightness, coincidence-to-accidental ratio (CAR), and heralding efficiency. Therefore, propagation loss effects on quantum light sources are crucial for studying quantum information technologies.

Here, we explore the effects of propagation loss on photon-pair generation in a lossy waveguide. A theoretical investigation of the loss effects was performed using the split-step method, and an experimental investigation was conducted in silicon waveguides. According to theoretical prediction, photon pairs with the highest brightness can be achieved at an optimum waveguide length. In addition, the heralding efficiency, CAR, and pair-generation bandwidth decrease with increasing length. To confirm the loss effects, we fabricated silicon waveguides of various lengths and tested the quality of the quantum light source. This study provides a path for developing efficient and high-quality on-chip quantum light sources for future complex quantum photonic technologies.

## 2 Theory

### 2.1 Theoretical modeling

The split-step method was adopted to analyze the photon pair generation process in a lossy waveguide [17]. This method makes it easy to model the SFWM generation and propagation loss simultaneously, which is difficult to handle the propagation loss of single photons in a lump sum. A waveguide of length (*L*) is split into (*M*) segments of length (*z* = *L*/*M*). In each segment, the SFWM process occurs, and the loss process follows. These processes are repeated *M* times while the pump light passes through a waveguide. The SFWM and optical loss operators of the *m*th segment are as follows [18–21]:

(1) $$U_{\mathrm{SFWM,}m}=1+\gamma P_{m}z{\text{e}}^{\text{i}\Delta kmz}{a_{s}}^{†}{a_{i}}^{†},$$

(2) $$U_{\text{loss},m}=\underset{q=s,i}{\prod }\left(e^{-\alpha z/2}{a_{q}}^{†}a_{q}+\sqrt{\alpha z}{b_{q,m}}^{†}a_{q}\right),$$

where ${a_{x}}^{†}$ and *a*_*x*_ are the photon creation and annihilation operators in a waveguide, respectively, and the subscripts *x* = {*P*, *s*, *i*} represent the pump, signal, and idler, respectively. *γ* and *P*_*m*_ are the Kerr coefficients of the waveguide and the pump power at the *m*th segment, respectively. The pump power at the *m*th segment is *P*_*m*+1_ = *P*_*m*_e^−*αz*^, and *P*_0_ is the initial pump power in the waveguide. The wavevector mismatch Δ*k* is Δ*k* = 2*k*_*P*_ − *k*_*s*_ − *k*_*i*_ − 2*γP*_*m*_, where *k*_*x*_ is the wavenumber. In our calculation, the influence of Kerr effect (2*γP*_*m*_), two-photon absorption, and free carrier absorption is sufficiently small below 2% under our experimental conditions. Therefore, we neglect the nonlinear loss and phase shift but consider the linear absorption loss *α* and Δ*k* = 2*k*_*P*_ − *k*_*s*_ − *k*_*i*_, to derive relatively simple equations for easy understanding and practical application. In addition, we ignored multiphoton pair events by assuming a low pump power. Eq. (2) is suitable for only one photon pair, and $\sqrt{1-e^{-\alpha z}}$ is approximated as $\sqrt{\alpha z}$ , due to a small length z of the segment. In Eq. (2), the optical loss in a segment of length *z* can be described as a beam splitter with two input ports (one for input photons and the other for vacuum) and two output ports (transmission and reflection). A photon in the reflection port can be represented by the creation operator ${b_{m}}^{†}$ , meaning that one photon of the photon pair is lost in the *m*th segment. The loss process for each segment is nonparametric and incoherent.

The operators function as follows for each Fock state:

(3) $$U_{\mathrm{SFWM,}m}\left|00\right\rangle \approx \gamma P_{m}z{\text{e}}^{\text{i}\Delta kmz}\left|11\right\rangle +\left|00\right\rangle .$$

(4) $$\begin{array}{ll} U_{\text{loss},m}\left|11\right\rangle & \approx e^{-\alpha z}\left|11\right\rangle +e^{-\alpha z/2}\sqrt{\alpha z}{\left|10\right\rangle }_{m}\\ & \;+e^{-\alpha z/2}\sqrt{\alpha z}{\left|01\right\rangle }_{m}. \end{array}$$

(5) $$U_{\text{loss},m}{\left|10\right\rangle }_{m^{\prime }}\approx e^{-\alpha z/2}{\left|10\right\rangle }_{m^{\prime }}.$$

The amplitude of the $\left|11\right\rangle$ state is much smaller than the $\left|00\right\rangle$ state as the pump power is small enough for ignoring multipair events. Therefore, the amount of transition from the $\left|11\right\rangle$ state to the $\left|00\right\rangle$ state is negligible compared to the existing $\left|00\right\rangle$ state. Therefore, the $\left|00\right\rangle$ terms were omitted in Eqs. (4) and (5).

Using the split-step method, the state of the photons passing through the *m*th segment is as follows:

(6) $$\left|\Psi \left(m\right)\right\rangle =\prod_{q=1}^{m}U_{\text{loss},q}U_{\mathrm{SFWM,}q}\left|\Psi \left(0\right)\right\rangle .$$

According to Eq. (6), the amplitude of a Fock state $\left|11\right\rangle$ through the waveguide length *L* is given by

(7) $$A_{11}\left(L\right)=\gamma P_{0}e^{-\alpha L}z\sum_{q=1}^{M}{\text{e}}^{\text{i}\Delta kqz}=\gamma P_{0}e^{-\alpha L}\int_{0}^{L}{\text{e}}^{\text{i}\Delta kz}dz.$$

Therefore, the coincidence-count rate of photon pairs through a lossy waveguide of length *L*, considering the filter’s spectral bandwidth (Δν) and the channel efficiencies of the signal (*η*_*s*_) and idler (*η*_*i*_) photons, is as follows:

(8) $$\begin{array}{ll} N_{CC} & =\Delta \nu \eta_{s}\eta_{i}{\left|A_{11}\left(L\right)\right|}^{2}\\ & =\Delta \nu \eta_{s}\eta_{i}{\left(\gamma P_{0}Le^{-\alpha L}\right)}^{2}{\mathrm{sinc}}^{2}\left(\frac{\Delta kL}{2}\right). \end{array}$$

Assume that an idler photon is annihilated in the *q*th segment and the correlated signal photon passes through the remaining segments. This process can occur somewhere between the beginning of the waveguide (*q* = 1) and its end (*q* = *M*). Therefore, the total probability of the idler’s extinction and the signal’s survival is given as follows:

(9) $$\begin{array}{ll} {\left|A_{10,total}\left(L\right)\right|}^{2} & =\overset{M}{\underset{q=1}{\sum }}{\left|A_{10,q}\left(L\right)\right|}^{2}\\ & =\int_{0}^{L}\alpha {\left|A_{11}\left(z\right)\right|}^{2}e^{-\alpha \left(L-z\right)}dz. \end{array}$$

Finally, the single-count rate through a lossy waveguide of length *L*, considering the filter’s bandwidth and channel efficiency, is given as follows

(10) $$\begin{array}{ll} N_{\text{SC},s} & =\Delta \nu \eta_{s}\left({\left|A_{11}\left(L\right)\right|}^{2},+,{\left|A_{10,total}\left(L\right)\right|}^{2}\right)\\ & =\Delta \nu \eta_{s}{\left(\gamma P_{0}Le^{-\alpha L}\right)}^{2}\\ & \;\times \frac{2\left(e^{\alpha L}-\frac{\alpha L}{\Delta kL}\sin\left(\Delta kL\right)-\cos\left(\Delta kL\right)\right)}{{\left(\alpha L\right)}^{2}+{\left(\Delta kL\right)}^{2}}. \end{array}$$

Here, the multipair generation events and other nonlinear effects, including two-photon absorption, are neglected, assuming low pump power. The single-count rate for the idler photons is similar to that in Eq. (10), except when using the idler photon’s channel efficiency (*η*_*i*_). *N*_CC_ is a value related to only the two-photon states, but *N*_SC_ contains not only the two-photon states but also the one-photon states, which is the lost state of one photon in a photon pair, yielding a slight difference between the single-photon and two-photon brightness. The theoretical equations of *N*_CC_ and *N*_SC_ are consistent with the existing theory [22–24], and we show the difference between them experimentally.

### 2.2 Performance evaluation parameters

#### 2.2.1 Brightness

Several parameters, including brightness, heralding efficiency, and CAR, have been used to evaluate the efficiency of a quantum light source in generating photon pairs [25, 26]. The coincidence-count brightness (*B*_CC_) and single-count brightness (*B*_SC_) are defined as $B_{\text{CC}}\equiv N_{\text{CC}}/\Delta \nu \eta_{s}\eta_{i}{P_{0}}^{2}$ and $B_{\text{SC}}\equiv N_{\text{SC},x}/\Delta \nu \eta_{x}{P_{0}}^{2}$ , respectively, to exclude the effects of filters, channel efficiencies, and pump power. According to Eqs. (8) and (10), *B*_CC_ and *B*_SC_ are as follows:

(11) $$B_{\text{CC}}={\left(\gamma Le^{-\alpha L}\right)}^{2}{\mathrm{sinc}}^{2}\left(\frac{\Delta kL}{2}\right),$$

(12) $$\begin{array}{ll} B_{\text{SC}} & ={\left(\gamma Le^{-\alpha L}\right)}^{2}\\ & \;\times \frac{2\left(e^{\alpha L}-\frac{\alpha L}{\Delta kL}\sin\left(\Delta kL\right)-\cos\left(\Delta kL\right)\right)}{{\left(\alpha L\right)}^{2}+{\left(\Delta kL\right)}^{2}}. \end{array}$$

The unit of brightness is Hz/mW^2^/nm. If the phase-mismatch parameter is small (Δ*kL* ∼ 0), the coincidence count brightness (*B*_CC_) in Eq. (11) has the maximum value of ${\left(\gamma /\alpha e\right)}^{2}$ at *L*_CC, max_ = 1/*α*. The single-count brightness (*B*_SC_) in Eq. (12) has a maximum value of approximately $1.5{\left(\gamma /\alpha e\right)}^{2}$ at *L*_SC, max_ ≈ 1.26/*α*. Note that the single-count brightness (*B*_SC_) contains contributions from both two-photon and one-photon states, yielding the maximum brightness at a slightly longer waveguide length for *B*_SC_ than for *B*_CC_.

#### 2.2.2 Heralding efficiency

Heralding efficiency, one of the parameters of the heralded single-photon scheme, has enormous effects on quantum experiments and applications requiring a large number of single photons and a high generation rate [27, 28]. The heralding efficiency is defined as *HE*_*x*_ ≡ *N*_CC_/*N*_SC,*x*_ and depends on the channel efficiency. The intrinsic heralding efficiency (*HE*_int_) was introduced to investigate the performance of a heralded single-photon source, excluding the channel efficiency, and is defined as *HE*_int,*x*_ ≡ *B*_CC_/*B*_SC,*x*_. For a negligible phase mismatch parameter Δ*kL* ≈ 0, the intrinsic heralding efficiency can be simplified as a function of *αL* as follows:

(13) $$HE_{\text{int}}\approx \frac{{\left(\alpha L\right)}^{2}}{2\left(e^{\alpha L}-\alpha L-1\right)}.$$

#### 2.2.3 Coincidence-to-accidental ratio

In quantum experiments, CAR affects measurement results, including quantum bit error rate, Hong–Ou–Mandel interferometer visibilities, and quantum state tomography fidelities [8, 29], and typically higher CAR values are preferred. Statistically, CAR can be expressed as follows:

(14) $$CAR=\frac{N_{\text{CC}}}{N_{\text{SC},s}N_{\text{SC},i}\Delta t},$$

where Δ*t* denotes the coincidence window. The measured *N*_SC,*x*_ includes dark counts, noise photons proportional to the pump power, and photons generated from the SFWM proportional to the square of the pump power [24, 30]. Assuming small dark counts, $N_{\text{SC}}=N_{\text{CC}}/HE+g\sqrt{N_{\text{CC}}}$ , where *g* is defined by the noise constant and is a constant related to the noise photons. Then, Eq. (14) becomes the following:

(15) $$CAR=\frac{N_{\text{CC}}}{{\left(N_{\text{CC}}/HE+g\sqrt{N_{\text{CC}}}\right)}^{2}\Delta t}.$$

## 3 Methods

### 3.1 Fabrication

**Figure 1:**
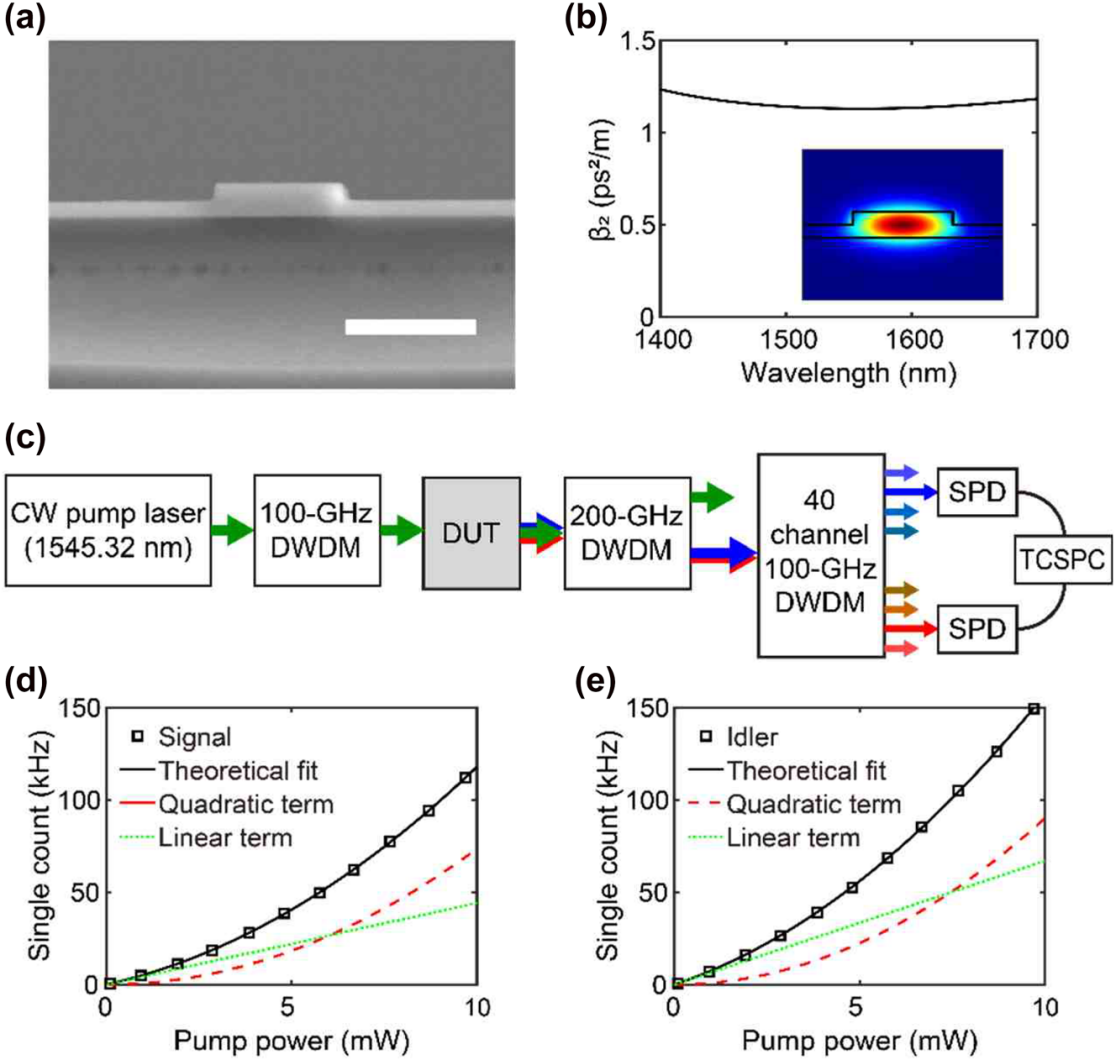
(a) Cross-sectional scanning electron microscope image of a fabricated waveguide. The scale bar is 1 μm. (b) Group velocity dispersion of the fabricated waveguide against wavelength. The lower right inset is the computed electric field profile of the optical transverse-electric-like mode. (c) Schematic diagram of the apparatus used to measure the properties of generated photon pairs. Components of the apparatus are labeled as follows: CW, continuous wave; DWDM, dense wavelength division multiplexing; DUT, device under test; SPD, single-photon detector; TCSPC, time-correlated single-photon counting circuits. (d) Measured single counts of signal photons against pump power. (e) Measured single counts of idler photons against pump power. The black squares in (d) and (e) are the experimental data, and the solid black lines are quadratic fittings of measured data. The dashed red and dotted green lines represent the quadratic and linear contributions of the fitting functions, respectively.

We fabricated several silicon photonic waveguides of various lengths to investigate the effects of propagation loss on photon-pair generation. In this study, we used a silicon-on-insulator platform. However, the investigation results were applicable to all material platforms using SFWM. The waveguides were patterned on a silicon-on-insulator chip with a silicon top layer of 260 nm and a buried oxide layer of 3 μm using KrF stepper photolithography. An inductively coupled plasma etcher with a mixture of C_4_F_8_ and SF_6_ was used to transfer the waveguide patterns to silicon. The etching depth was set as 130 nm. Then, a SiO_2_ layer was deposited on the chip as a top cladding by 1.4 μm. A cross-sectional scanning electron microscope image of the fabricated waveguide is shown in Figure 1(a). The waveguide width is 1 μm, and the waveguide lengths are 0.8 cm, 1.4 cm, 2.0 cm, 3.2 cm, 4.4 cm, and 5.6 cm. The measured absorption coefficient *α* is about 0.51 cm^−1^ ( ≈ 2.22 dB/cm). Grating couplers are used to couple light in and out of a waveguide to a single-mode fiber.

### 3.2 Simulation

COMSOL Multiphysics software was used to estimate the wavevector mismatch (Δ*k*) using the mode-eigenvalue solver based on the finite element method. Figure 1(b) shows the group velocity dispersion (*β*_2_) extracted from the numerical calculations. *β*_2_ is 1.13 ps^2^/m and is almost constant over the C-band. Note that a zero-GVD wavelength does not exist in the telecom band, as shown in Figure 1(b). It is known that photon-pair generation with a small detuned frequency from the pump frequency occurs near the zero GVD wavelength. However, our recent results show that if the phase-mismatch parameter Δ*kL* is less than 1 [31], photon pairs can be created in an optical fiber, regardless of the zero GVD wavelength. If we apply this concept to silicon photonic waveguides, photon pairs can be generated even in a waveguide at a frequency far from the zero GVD wavelength. Therefore, a rib-type waveguide can be a photon-pair source as long as the waveguide length is sufficiently short, even if it has no zero GVD wavelength. Because a rib-type waveguide typically has less scattering loss than a ridge-type waveguide, the use of rib-type optical waveguides is advantageous in quantum optics experiments.

Using the second-order Taylor approximation [24], the wavevector mismatch is approximately

(16) $$\Delta k=2k\left(f_{P}\right)-k\left(f_{P}+df\right)-k\left(f_{P}-df\right)\approx -4\pi^{2}\beta_{2}df^{2},$$

where d*f* is the detuned frequency relative to the pump frequency ( $df=\left|f_{P}-f_{s}\right|=\left|f_{P}-f_{i}\right|$ ) and *f*_*x*_ is the frequency of pump, signal, or idler photons. Assuming that the detuned frequency of the signal and idler photons from the pump frequency is d*f* = 300 GHz, the calculated wavevector mismatch is Δ*k* = 4.01 m^−1^. Then, photon pairs can be generated in the fabricated silicon waveguides with a length of dozens of centimeters. The inset of Figure 1(b) shows the fundamental transverse electric-like mode in a waveguide with the same waveguide geometry as shown in Figure 1(a).

### 3.3 Experiment

A schematic of the experimental setup is shown in Figure 1(c). The pump light source was a continuous-wave laser operating at 1545.32 nm, and a series of 100-GHz dense wavelength division multiplexing (DWDM) filters suppressed the sideband noise photons of the pump. The pump light passes through the device under test, and then a series of notch filters (200 GHz DWDM filters) filters out the pump from the generated photon pairs. Then, the signal photons are spectrally separated from the idler photons using a 40-channel 100-GHz DWDM module. The bandwidth Δ*ν* of the DWDM module was 0.6 nm. Photons were detected using superconducting nanowire single-photon detectors (Scontel). The estimated channel efficiencies of the signal and idler photons were approximately −15.5 and −14.7 dB, respectively. A time-correlated single-photon counting (TCSPC) module was used for the coincidence count measurements.

In addition to the generated signal and idler photons, single-photon detectors detect noise photons, including Raman-scattered photons, pump leakages, and dark counts. The single-count rate of each detector can be described as a quadratic function of pump power [30]. The quadratic term for the pump power is related to the generated signal or idler photons, whereas the noise photons contribute to the linear term. The dark counts of the detectors did not correlate with the pump power. The square markers in Figure 1(d) and (e) are the measured signal and idler single-count rates against the pump power, respectively. The theoretical fits were obtained using the quadratic function of the pump power and are shown as solid black curves superimposed on the data. The quadratic and linear contributions of the fitting functions corresponding to pair generation and noise photons are shown as dashed red and dotted green curves, respectively. The dark count rates were approximately 100 Hz and were negligible. Using the quadratic contributions of the fitting functions, we can estimate the contribution of the generated signal or idler photons to single-count rates, removing the contributions of noise photons and dark counts in the raw single-photon counts. However, CAR is affected by the contributions of noise photons according to Eq. (15).

## 4 Results

### 4.1 Length dependence

**Figure 2:**
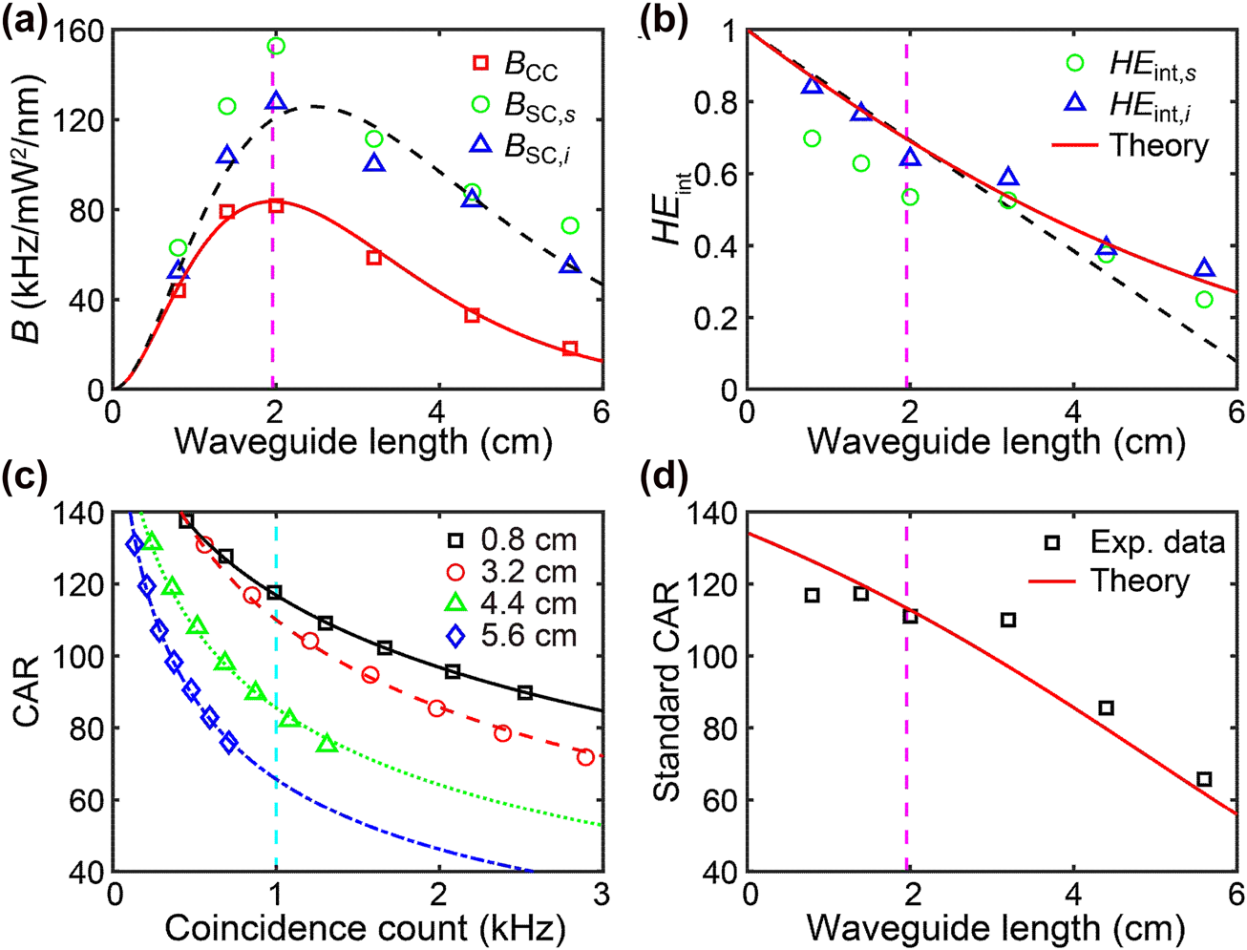
Measured brightness and CAR with a 300-GHz detuned frequency. (a) Brightness against the waveguide length. The red squares (*B*_CC_), green circles (*B*_SC,*s*_), and blue triangles (*B*_SC,*i*_) are the experimental data. The theoretical fit using Eqs. (11) and (12) are shown as solid red and dashed black lines, respectively, atop the experimental data. (b) The intrinsic heralding efficiency against the waveguide length. The green circles (*HE*_int,*s*_) and blue triangles (*HE*_int,*i*_) are the experimental data. The theoretical fit using Eq. (13) is shown as solid red atop the experimental data, and the dashed black curve is the linear approximation of *HE*_int_ ≈ 1 − 0.3*αL* for *L* ≤ 1/*α*. (c) The CAR against the measured coincidence counts for various waveguide lengths [0.8 cm, 3.2 cm, 4.4 cm, and 5.6 cm]. The symbols are the experimental data for various waveguide lengths. The theoretical fit using Eq. (15) is shown as solid black, dashed red, dotted green, and dashed-dotted blue atop the experimental data. The vertical dashed cyan line shows where *N*_CC_ = 1 kHz. (d) Standard CAR against the waveguide length. The black square represents experimental data. The theoretical fit (solid red) is obtained using Eq. (15) with *g* = 3972 HZ^1/2^ and the extracted CAR value at *N*_CC_ = 1 kHz in (c). The dashed magenta lines in (a), (b), and (d) show where *L* = 1/*α* ≈ 1.96 cm.

The experimental results for brightness, heralding efficiency, and coincidence-to-accidental ratio are shown in Figure 2. From the simulation results for *β*_2_ and Eq. (16), the calculated wavevector mismatch is Δ*k* = 4.01 m^−1^ with d*f* = 300 GHz, and the phase mismatch parameter can be neglected (Δ*kL* ∼ 0) by assuming a short waveguide *L* ≪ 1 m. From the measured absorption coefficient *α* = 0.51 cm^−1^, the waveguide length for the maximum coincidence-count brightness is *L*_max_ = 1.96 cm and as shown by the magenta dashed lines in Figure 2. The red square markers in Figure 2(a) are the measured coincidence-count brightness (*B*_CC_), and the *B*_CC_ value has a maximum value of 82 kHz/mW^2^/nm at a 2.0-cm ( ∼1/α ) long waveguide. Kerr coefficient *γ* ≈ 114 W^−1^ m^−1^ is obtained from the *γ* value where the experimental data of *B*_CC_, *B*_SC,*s*_, and *B*_SC,*i*_ fit well to Eq. (11) and (12). The green circle and blue triangle markers in Figure 2(a) represent the measured single-count brightness of the signal (*B*_SC,*s*_) and idler (*B*_SC,*i*_) photons, respectively. From Eq. (12), the expected single-count brightness (*B*_SC_) is shown as a dashed black curve in Figure 2(a) and has a maximum value of 126 kHz/mW^2^/nm at a 2.5-cm ( ∼1.26/α ) long waveguide.

According to Eq. (13), the heralding efficiency *HE*_int_ decreases as *αL* increases, as shown by the solid red curve in Figure 2(b). At *L* = 1/*α*, where *B*_CC_ has the maximum value, the expected *HE*_int_ ≈ 0.7. The measured intrinsic heralding efficiencies are 0.53 and 0.64 at a 2.0-cm long waveguide for signal and idler photons, respectively. The measured values differ from the theoretical expectation owing to fabrication and measurement errors, but the overall data trend follows the theory. When *L* ≤ 1/*α*, the intrinsic heralding efficiency can be approximated by a simple equation as *HE*_int_ ≈ 1 − 0.3*αL*, as shown by the dashed black curve in Figure 2(b).

Figure 2(c) shows that CAR generally decreases as *N*_CC_ and waveguide length increase. According to Eq. (15), CAR strongly depends on *N*_CC_, and a high CAR value can be obtained at a low *N*_CC_. Because such a low *N*_CC_ is not practical for quantum experiments, we define the CAR value at *N*_CC_ = 1 kHz, which is widely used in this study, as the standard CAR (vertical dashed cyan line) in Figure 2(c). In Eq. (15), the CAR is also affected by *HE*_int_ and *g*. The *g* value was extracted from the fitting results and was approximately 3972 Hz^1^ ^/2^. Figure 2(d) shows the experimental data of the standard CAR against the waveguide length owing to the influence of *HE*_int_, which matches well with the theoretical value. This result shows that the waveguide length affects not only *HE*_int_ but also the standard CAR.

### 4.2 Frequency dependence

**Figure 3:**
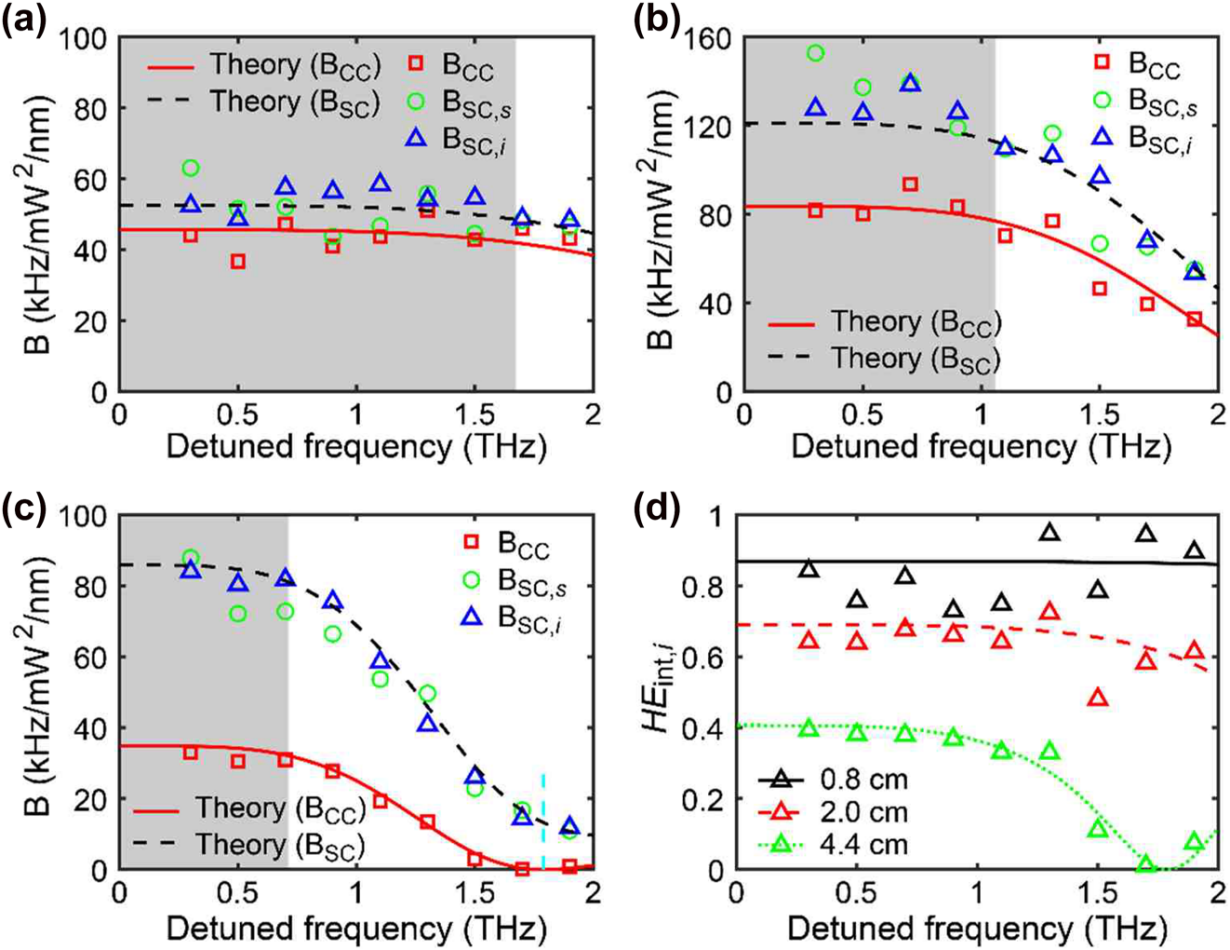
Brightness against the detuned frequency for the waveguide lengths of (a) 0.8 cm, (b) 2.0 cm, and (c) 4.4 cm. The red squares (*B*_CC_), green circles (*B*_SC,*s*_), and blue triangles (*B*_SC,*i*_) are the experimental data. The theoretical fit using Eqs. (11) and (12) are shown as solid red and dashed black lines, respectively, atop the experimental data. The vertical dashed cyan line in (c) shows where Δ*kL* = 2*π* for *L* = 4.4 cm, and the grey regions represent where the detuned frequency for Δ*kL* < 1. (d) Intrinsic heralding efficiency of idler photons versus the detuned frequency for various waveguide lengths. The black, red, and green triangles (*HE*_int,*i*_) are the experimental data for *L* = 0.8 cm, 2.0 cm, and 4.4 cm, respectively. The theoretical fits for various lengths are shown atop the experimental data.

Figure 3 shows the brightness and *HE*_int_ against the detuned frequency for various waveguide lengths. According to Eqs. (11) and (12), *B*_CC_ and *B*_SC_ vary slowly when Δ*kL* < 1, and the *B*_CC_ value at Δ*kL* = 1 can be obtained with over 92% of the maximum *B*_CC_, as shown in Figure 3(a). The slightly varying *B*_CC_ when Δ*kL* < 1 is similar for all waveguide lengths, as shown in Figure 3(b) and (c). The detuned frequency d*f* for Δ*kL* < 1 is indicated by the gray region in Figure 3. As the waveguide length increases, the spectral bandwidth for Δ*kL* < 1 decreases. This result is consistent with our recent results for optical fibers [31].

In Figure 3(c), the coincidence count brightness *B*_CC_ is zero when d*f* ≈ 1.79 THz, where Δ*kL* = 2*π*. However, the single-count brightness *B*_SC_ is not zero because this phenomenon is related to the two-photon and one-photon states. The generation of two-photon states by SFWM is a parametric process that requires conservation of energy and momentum (phase-matching) conservation. From the phase-matching conditions, the probability of having two photons is zero when Δ*kL* = 2*π*. On the other hand, the one-photon states are caused by generating a pair of photons and losing one photon out of them, and the photon loss process is a nonparametric process that is not affected by the phase. Therefore, *B*_SC_ does not become zero when Δ*kL* = 2π.

Figure 3(d) shows that the spectral bandwidth of the signal photon *HE*_int,*i*_ decreased as the waveguide length increased. The spectrum of *HE*_int,*s*_ is similar to that of *HE*_int,*i*_. The reduction in *HE*_int,*i*_ is negligible when Δ*kL* < 1, where *B*_CC_ is efficient.

## 5 Conclusions

We investigated the effects of propagation loss on photon-pair generation in a lossy waveguide. The theoretical modeling approach is performed through the split-step method, in which the SFWM and photon loss processes are repeated. Silicon waveguides of various lengths were fabricated as quantum light sources to experimentally examine the effects of propagation loss on the performances of photon pairs, including brightness, heralding efficiency, and CAR. The theoretical modeling results agreed well with the measurement results. The brightness increases (decreases) as the waveguide length increases for *L* < 1/*α* (*L* > 1/*α*), and the coincidence-count brightness has a maximum value at *L* = 1/*α*. In addition, the intrinsic heralding efficiency was approximated as *HE*_int_ ≈ 1 − 0.3*αL*. With a small phase-mismatch parameter Δ*kL* < 1, photon pairs can generate more than 92% of the maximum brightness and undiminished intrinsic heralding efficiency. The above results show a trade-off relationship between heralding efficiency and brightness against length, but the optimized length for an experiment may differ depending on the application field and how the on-chip quantum light source is used. The results obtained in this study on the propagation loss effect of photon pair generation in a lossy waveguide provide information necessary for the practical development of on-chip quantum light sources and provide expectations for the output performance of a fabricated quantum light source. We believe that this study will be helpful in the development of practical quantum light sources for on-chip-based quantum applications, such as quantum computing, quantum teleportation, and quantum key distribution.
